# Qualitative Study of Breast Cancer and Lymphoma Patients Participating in a Physical Activity Intervention: The Case for Tailored Physical Activity

**DOI:** 10.1002/cam4.71747

**Published:** 2026-03-24

**Authors:** Brianna N. Leitzelar, Juliana V. Costa, Aylin A. Aguilar, Brianna R. Wolle, Alexander R. Lucas, Alexandra Marshall, Sam Norton, Shannon L. Mihalko, Peter H. Brubaker, Rakhee Vaidya, Mary Beth Seegars, Victor Yazbeck, R. Lee Franco, Jeremy Via, W. Gregory Hundley, Lynne I. Wagner

**Affiliations:** ^1^ Department of Social Sciences and Health Policy Wake Forest University School of Medicine Winston‐Salem North Carolina USA; ^2^ School of Kinesiology University of Minnesota‐Twin Cities Minneapolis Minnesota USA; ^3^ Department of Health and Exercise Science Worrell Professional Center, Wake Forest University Winston‐Salem North Carolina USA; ^4^ Qualitative and Patient‐Reported Outcomes Shared Resource Atrium Health Wake Forest Baptist Comprehensive Cancer Center Winston‐Salem North Carolina USA; ^5^ Department of Internal Medicine—Cardiology Virginia Commonwealth University Richmond Virginia USA; ^6^ Department of Social and Behavioral Sciences Virginia Commonwealth University School of Public Health Richmond Virginia USA; ^7^ Department of Internal Medicine (Section on Hematology‐Oncology) Wake Forest University School of Medicine Winston‐Salem North Carolina USA; ^8^ Massey Cancer Center Virginia Commonwealth University Richmond Virginia USA; ^9^ Department of Internal Medicine Virginia Commonwealth University Richmond Virginia USA; ^10^ Department of Kinesiology and Health Sciences Virginia Commonwealth University Richmond Virginia USA; ^11^ Department of Health Policy and Management University of North Carolina Gillings School of Global Public Health Chapel Hill North Carolina USA

**Keywords:** breast cancer, cardiotoxicity, exercise, lymphoma, participant perspectives, qualitative research

## Abstract

**Background:**

Exercise mitigates chemotherapy‐related cardiotoxicity, but implementing exercise interventions during chemotherapy treatment can be challenging. Understanding the patient perspective can help refine exercise programs and facilitate successful implementation of future interventions.

**Purpose:**

To utilize qualitative data to optimize adherence and retention for a future randomized clinical trial involving a tailored physical activity (PA) intervention among breast cancer and lymphoma patients receiving potentially cardiotoxic chemotherapy.

**Methods:**

Eighteen breast cancer and lymphoma patients receiving potentially cardiotoxic chemotherapy who enrolled in a PA intervention completed semi‐structured interviews. Interviews focused on reasons for enrollment or dropout, perceptions of study personnel, experiences within the study interventions, and barriers to intervention adherence. Interviews were audio recorded. Key points and potential themes were identified using an integrative synthesis of the data.

**Results:**

Participants were on average 53 years (SD = 15) old, were 50% female (*n* = 9), predominantly identified as White (*n* = 15, 83%), and were mostly non‐Hodgkin Lymphoma patients (*n* = 11, 61%). The main reasons for enrollment were altruism and provider recommendation. Participants stated they completed the study because of intrinsic motivation, ease of participation, and positive study experiences. Participants reported favorable perceptions of the study interventions and study staff. Barriers to adherence included symptom burden and lack of motivation.

**Conclusions:**

Messages to enhance altruism and collaboration with providers may be important recruitment tools. Developing positive study‐staff relationships and delivering tailored, flexible interventions may enhance study adherence and retention. Ultimately, these strategies contribute to the successful implementation of cancer exercise trials.

**Trials Registration:**

NCT01719562

## 1 Introduction

Effective strategies to reduce or prevent chemotherapy‐related cardiotoxicity may be critical to maintain or promote health and wellbeing throughout cancer treatment into survivorship [1, 2, 3]. Exercise mitigates the cardiotoxic effects of chemotherapy treatment among breast cancer and lymphoma survivors [4, 5, 6, 7, 8, 9, 10, 11]. Specifically, 8–12 weeks of aerobic or combined aerobic and resistance exercise training, compared to usual care, improves cardiorespiratory fitness [5, 6, 7, 8, 9] and prevents declines in cardiovascular health [6, 10]. Additional benefits of exercise for cancer survivors range from improved mortality and reduced risk for cancer recurrence [12, 13, 14] to acute physiological adaptations [15, 16, 17], highlighting the importance of engaging in exercise for cancer survivors.

Implementing exercise regimens can be challenging, particularly for patients experiencing acute effects of chemotherapy [18, 19]. Exercise initiation and adherence during anthracycline treatment are problematic due to cancer therapy‐associated fatigue, nausea, and muscle weakness. For example, in a clinical exercise trial among lymphoma patients [9], ~20% of screened participants were not eligible due to symptom and health‐related reasons (treatment adverse effects, serious conditions, frailty), and the most common reason for declining study participation included disinterest or no reason (20%). Understanding patient experiences, namely their interests, perspectives, barriers, and facilitators for participation, can contribute to understanding how to refine exercise programs best to optimize adherence and retention.

The Physical Activity and Lifestyle Study's (PALS) innovative design utilized qualitative research methodology to tailor the delivery of an individualized exercise regimen based on patient input. During the initial feasibility pilot phase, breast cancer and lymphoma patients were randomized to physical activity or healthy living control interventions. Our team sought participant feedback on reasons for enrollment, perspectives of the programs, and barriers to adherence with the goal of optimizing adherence and retention for a subsequent RCT, and ultimately to inform strategies to promote physical activity adherence in clinical practice. The purpose of this paper is to describe our qualitative findings and provide insight into how they were used to refine intervention design for the subsequent larger‐scale RCT.

## 2 Materials and Methods

The Wake Forest University Institutional Review Board provided institutional ethics approval (IRB00020968), and all participants provided written or verbal informed consent. This was a qualitative study of participants' perspectives while participating in the PALS pilot RCT. PALS was conducted at Atrium Health Wake Forest Baptist Medical Center (AHWFBMC) and Virginia Commonwealth University Masssey Comprehensive Cancer Center (VCUMCCC). The following describes methods pertinent to the qualitative component; detailed methods for the PALS pilot RCT are reported elsewhere [20]. In brief, eligible participants were randomly assigned to a 6‐month physical activity intervention (PAI) or a health living intervention (HLI) control. Participants completed assessments at pre‐intervention (baseline), mid‐point (3‐months), and post‐intervention (6‐months). The qualitative study was initiated approximately 1 year following the initiation of PALS, and interviews were conducted between May 2020 and March 2022. Participants who completed the study were invited on a rolling basis and were contacted about 4 weeks after their 6‐month follow‐up visit (±1 week) or earlier if participants stopped attending PAI or HLI sessions.

### 2.1 Study Population

Individuals eligible for PALS were aged 18–85 years, diagnosed with breast cancer or non‐Hodgkin or Hodgkin lymphoma, within 8 weeks of completing potentially cardiotoxic cancer therapies (e.g., anthracyclines, immune‐checkpoint inhibitors, or radiation), able to walk ≥ 2 city blocks, and spoke English. Exclusion criteria included: (1) uncontrolled hypertension, (2) recent substance abuse, (3) presence of an inflammatory condition, (4) contraindications to MRI, (5) unstable angina, (6) inability to exercise on a treadmill or stationary bicycle, (7) significant ventricular arrhythmias, (8) atrial fibrillation with uncontrolled ventricular response, (9) acute myocardial infarction within 28 days, or (10) moving within 12 months of enrollment.

### 2.2 Interventions

#### 2.2.1 PAI

Participants assigned to the physical activity intervention group completed a tailored 6‐month progressive exercise training program. The program consisted of two 60‐min sessions per week of supervised progressive moderate‐intensity aerobic and resistance training. Participants could choose their preferred mode of aerobic exercise (e.g., treadmill walking, bicycling, etc.). Initially, intensity began at 50% of one's maximal cardiorespiratory fitness (VO_2 peak_), progressing up to 75% of VO_2 peak_. In addition to structured exercise, participants were encouraged to engage in lifestyle physical activity (≥ 30 min/day of light‐moderate intensity exercise).

Supervised sessions were delivered in‐person by a clinical exercise psychologist. Exercise programs were tailored to individual functional capacities and modified by participants' daily perceived levels of treatment‐related symptoms (i.e., fatigue) following a nonlinear approach [21]. Adherence and retention to PAI were facilitated by a flexible toolbox of behavioral techniques, which included frequent contact with participants, positive feedback, goal achievement incentives, promoting community, enhancing self‐efficacy and outcome expectations, and teaching self‐regulatory skills and problem‐solving. Participants were given a FitBit activity tracker in order to self‐monitor their PA behavior.

In‐person supervised sessions were adapted for the home environment for the following cases. Participants who could not travel for in‐person sessions completed their individualized exercise program at home. Completion of the home‐based program was monitored using data obtained from a Fitbit activity tracker and through discussions between the interventionists and participants. At the onset of the global COVID‐19 pandemic, all supervised exercise sessions were delivered remotely using the online Zoom platform. Once it was deemed safe, participants were offered a choice between in‐person and home‐based exercise sessions.

#### 2.2.2 HLI

Participants assigned to the control intervention attended 60‐min group‐based health education sessions approximately every 2 weeks. The purpose of HLI was to control for staff attention and social interaction within the intervention group and was not expected to influence the primary outcome of the study. HLI topics included proper nutrition, stress management, sleep, finances, blood pressure, wellness, social support, pain management, and transitioning to life after treatment. Each session ended with 10 min of light stretching. Similar to PAI, HLI sessions were initially held in‐person but were then moved to the online Zoom platform due to the COVID‐19 pandemic. Virtual sessions allowed participants to connect across both study sites. As a result of this added social connection, sessions remained online for the rest of the study, even after COVID‐19 restrictions were lifted.

### 2.3 Data Collection

Qualitative Researchers from the Qualitative and Patient‐Reported Outcomes (Q‐PRO) Shared Resource at the AHWFBMC collected and analyzed the data. Collaborating with Q‐PRO for the qualitative data collection mitigated potential positive bias in the results, as the qualitative researchers were independent of the PALS study team and were not involved in the initial recruitment, assessment, nor intervention methods. A member of the PALS team provided participant contact, participant sex (female/male), and group assignment to a member of the Q‐PRO team. A member of the Q‐PRO team contacted PALS participants. Interviews were conducted over the telephone within approximately 4 work weeks (mean = 8.6 days, SD = 8.4 days). Interviewers followed a semi‐structured guide designed to elicit feedback about study participation to understand reasons for enrollment and continued participation or dropout (recruitment and retention), perceptions of study personnel, experiences within the study interventions (if expectations were met and perceptions of the programs), and feedback on study assessments (Table 1). All interviews were audio‐recorded, and the interviewer kept detailed written notes about each interview.

**TABLE 1:** Main interview questions.

Recruitment and retention	When you first learned about PALS, what were the reasons that you wanted to participate? For each reason, how did we meet or fail that expectation? 3a. *Non‐completers only*: What is your primary reason for leaving the study early? 3b. *Completers only*: What was the primary reason that you were successful in completing the entire 6‐month requirement?
Experience with study personnel	1. Tell me about your experience with the PALS study staff at the exercise sessions. 2. In what specific areas do you think we can improve PALS in terms of staffing, training or participant follow‐up?
Experience in study interventions	1a. *PAI only*: What do you think about the [exercise] sessions and the way they were designed? How can PALS improve upon the exercise sessions? 1b. *HLI only*: What do you think about the content, delivery and format of the health workshops? 2. If you feel that you benefitted from PALS in ways we haven't already discussed, emotionally or physically, tell me about those benefits. 3. Are there any other suggestions you can offer, in any area of the program, to make PALS better?

### 2.4 Data Analysis

Descriptive data were summarized and reported using summary statistics. Written interviewer notes served as the primary data source and original audio recordings were re‐reviewed to facilitate analysis. The written notes were transcribed, incorporating additional details referenced from the audio recordings. Subsequently, the audio files were transcribed verbatim. Interviewer notes and audio transcripts were reviewed concurrently, and additional detail was added to interviewer notes as needed. Data were stored on a secure server and managed using Microsoft Excel.

The present analysis aimed to identify key points, potential themes, and areas of further exploration. Consequently, data were not formally coded; instead, a matrix analysis was conducted. This approach was deemed appropriate due to the structured nature of the interview guide. Questions and answers were arranged in matrix form and data were summarized and synthesized using a thematic analysis approach. This paper provides an integrative summary and synthesis of the data. Preliminary results were discussed with the study team and data analysis was refined until consensus was reached (i.e., triangulation). The study team kept a record of these discussions and refinements (i.e., audit trail).

## 3 Results

### 3.1 Participant Characteristics

Thirty‐one patients with either breast cancer (*n* = 9) or lymphoma (*n* = 22) enrolled in the PALS study. Of those, thirteen participants did not complete the semi‐structured interview (*n* = 10 lost to follow‐up, *n* = 1 deceased, *n* = 2 declined). Eighteen participants (58%) completed the semi‐structured interview and comprised the participant sample for the qualitative study. Of the 18, 3 individuals were classified as non‐completes as they withdrew prior to PALS study completion, 9 individuals completed the PAI program, and 6 completed the HLI program. Mean age of interviewed participants was 53.1 years (SD = 14.9); 50% were female (*n* = 9), and the majority identified as White and were diagnosed with non‐Hodgkin lymphoma (Table 2).

**TABLE 2:** Participant characteristics.

Participant identifier	Group assignment	Completer	Age (years)	Sex	Race	Cancer type	Time between end of study participation and QPRO interview (days)
1P‐N	PAI	N	41	M	White	Hodgkin lymphoma	NA^a^
2P‐N	PAI	N	61	F	White	Non‐Hodgkin lymphoma	NA^a^
3P‐N	PAI	N	54	F	White	Non‐Hodgkin lymphoma	NA^a^
4P‐Y	PAI	Y	37	F	White	Breast	17
5P‐Y	PAI	Y	63	F	White	Non‐Hodgkin lymphoma	3
6P‐Y	PAI	Y	70	M	White	Non‐Hodgkin lymphoma	23
7P‐Y	PAI	Y	58	M	White	Non‐Hodgkin lymphoma	23
8P‐Y	PAI	Y	39	M	White	Non‐Hodgkin lymphoma	16
9P‐Y	PAI	Y	53	F	White	Breast	3
10P‐Y	PAI	Y	75	M	White	Non‐Hodgkin lymphoma	42
11P‐Y	PAI	Y	23	M	White	Non‐Hodgkin lymphoma	39
12P‐Y	PAI	Y	38	F	Black	Hodgkin lymphoma	7
13H‐Y	HLI	Y	40	M	Asian	Hodgkin lymphoma	655^b^
14H‐Y	HLI	Y	53	F	Asian	Breast	42
15H‐Y	HLI	Y	68	M	White	Hodgkin lymphoma	3
16H‐Y	HLI	Y	61	F	White	Non‐Hodgkin lymphoma	430^b^
17H‐Y	HLI	Y	45	F	White	Non‐Hodgkin lymphoma	37
18H‐Y	HLI	Y	77	M	White	Non‐Hodgkin lymphoma	18

Abbreviations: HLI, healthy living intervention; PAI, physical activity intervention.

^a^ Participant withdrew before the end of the study; data not available.

^b^ Participant completed PALS before qualitative interviews started but still agreed to complete the interview.

### 3.2 Recruitment and Retention

Data are organized by reasons for enrolling, completing, and withdrawing from the study. Participants could provide multiple data points per question; therefore, percentages may not total 100%. Explanatory quotes are in Table 3.

**TABLE 3:** Explanatory quotes derived from detailed interviewer notes.

Recruitment and retention	Study enrollment	“…wanted to contribute to research and wanted the findings of this study to help others with cancer.” (9P‐Y)
		“interested in participating in PALS because [they] wanted to know what was going on with [their] body and health, following [their] breast cancer diagnosis, and how [their] cancer treatment will affect [their] heart. …interested in the MRI testing and the effect of the stretching exercises, in particular.” (14H‐Y)
		“interested in participating in PALS because (1) [they] works in science and values and wants to contribute to research, and (2) [they] was unsure about how [they] would feel during her cancer treatment and wanted to participate in PALS for the accountability and motivation to move.” (4P‐Y)
		“… liked that the program did not seem too onerous or unusual in what was asked of participants…thought that it would be helpful for [them].” (13H‐Y)
		“…doctor told [them] it would help [them] recover from some…cancer treatments, and…thought it would motivate [them] to get up and get out of the house.” (1P‐N)
		“…heard about the study from [their] oncologist…always wanted to help others…” (16H‐Y)
	Study completion	“…also attributed [them] completing the study due to the friendly staff.” (12P‐Y)
		“…was successful at completing the full 6‐month program because of the proximity of the health workshop facility to [their] home, as well as the follow up from staff and the support that [they] received from both study staff and [their partner] to complete the study.” (13H‐Y)
		“…had the desire to complete the program…attributed to [their] perception that the quality of the program was good.” (13H‐Y)
		“…it gave [them] a routine… also said the study gave [them] a sense of fulfillment.” (11P‐Y)
		“…completed the study, because [they] finishes what [they] starts…the study staff was flexible, which also helped [them] continue the study….” (16H‐Y)
	Study withdrawal	“…went back to work after finishing [their] cancer treatment…did not have the energy to exercise after work.” (1P‐N)
		“…not motivated to do the exercises [them]self…would have benefited from doing the exercises in a group.” (2P‐N)
		“…did not have the space or equipment to exercise at home.” (2P‐N)
		“…had trouble understanding the technology.” (2P‐N)
		“…felt that [they] did not have extra time to attend appointments or do testing for PALS when [they] was in town for [their] treatment…not interested in traveling to the hospital for additional appointments.” (3P‐N)
Experience in PAI	Expectations met	“…the program met [their]expectations in that…monitored throughout the study and was provided with tests, like an MRI, that gave [them] more insight about [them]self.” (9P‐Y)
		“…was not expecting the study to change [their] life, and it didn't, but rather reinforced behaviors [they] already practiced.” (7P‐Y)
		“…the study met [their] expectations of staying more active ‘through and through.’ … because [they] had access to a facility and a study member that would help him.” (11P‐Y)
		“…hoping to have [their] heart health monitored throughout [their] cancer treatment, and felt that this expectation was met at the beginning of the study, when [they] was closely monitored through in‐person exercises.” (5P‐Y)
		“…enjoyed socializing with study staff at exercise sessions, and sessions were easy to schedule and attend at his convenience…goal was to attend 3 times per week, but he missed a few sessions due to medical appointments and recovery from a fall.” (6P‐Y)
		“…did really well in the first three months but then waned, because of treatment side effects and a lack of personal motivation…had in person workout session been possible, this definitely would have helped with [their] motivation.” (7P‐Y)
		“…unexpected benefit of the study for [them] was the ability to connect with other participants/cancer patients via the Zoom calls…appreciated hearing from others who were further along in their treatment about what to expect and thought the connection was a ‘tremendous positive.’…” (7P‐Y)
		“…liked them a lot, because they helped [them] get in better shape…also liked that [they] had creative freedom, while still getting help from a study team member…overall the sessions were easy, but some days were hard according to how [they] was feeling physically due to treatment.” (11P‐Y)
		“…it was challenging to exercise at the beginning of the program, because [they] would have chemotherapy twice every…month…noticed that the more [they] worked out, the more it helped [them] with fatigue and [their] chemotherapy brain.” (12P‐Y)
		“…valued the interaction with other participants, liked being a helpful resource to them, and thought the calls were important…described the calls as an “emotional mixed bag,” because it could be depressing to hear about others' circumstances, which were mostly worse than [their] own.” (8P‐Y)
		“…only challenge with the exercise sessions was getting there…it was a 35 min drive for [them] to get to [City], and then another 35 min drive after the session to go back to work…” (5P‐Y)
Experience in HLI	Expectations met	“…did not have clear expectations for the program…the study has (positively) surprised [them] so far, as [they] has enjoyed the Zoom workshops with another study participant more than [they] expected to.” (17H‐Y)
		“… the program exceeded [their] expectations, and praised the study staff that [they] interacted with for making it a good experience.” (13H‐Y)
		“…needed a physical and a mental outlet following [their] treatment, which PALS met.” (15H‐Y)
		“…health workshops have been excellent, that [they] has received good information from the workshops, and that the point people are doing an excellent job…particularly the ones on diabetes, cholesterol, and meditation—which [they] practices 5 days per week. …The participant said that the video conferencing has been good…although [they] likes being able to interact with the study team in person.” (14H‐Y)
		“…particularly liked the content on meditation and blood pressure….the study team works with the participants around their schedules…Zoom has been convenient, particularly with the COVID‐19 pandemic, but also because [they] does not have to drive to the workshops so it takes less time, and because [they] can still attend if [they] is out of town.” (17H‐Y)
		“…liked having the workshops over Zoom because [they] does not live close to the facility, and driving in for in‐person workshops would have been harder….” (15H‐Y)
		“…liked the interactive portion of the workshops—the stretching exercises—and thought that the information content was good and holistic.” (13H‐Y)
		“after the session on sleep [they] used some of the ideas presented and got better sleep.” (18H‐Y)
		“[h]ard of hearing sometimes [they] had difficulties…think that Zoom was more difficult than live would have been.” (18H‐Y)
		“…the health workshops went well, and that [they] appreciated the interaction and information…particularly enjoyed the tips for combatting cancer side effects, as well as the content on nutrition…was a big help….” (15H‐Y)
		“…liked that [they] was able to have a group were [they] could be open…the most helpful [topic] was the mental health one…other topics, such as nutrition, were helpful; however, they contained information [they] already knew….” (16H‐Y)

*Note:* The primary source of data was detailed field notes from interviewers, thus they are written in third person. Gender‐identifying language has been edited to be gender neutral in order to maintain participant privacy.

#### 3.2.1 Reasons for Study Enrollment

Altruism (*n* = 8, 44.4%) was given as the primary reason for enrollment. The second‐most endorsed reason was due to provider recommendation (*n* = 6; 33.3%). Other reasons included personal investment (*n* = 5, 27.8%), health monitoring (*n* = 5, 27.8%), the potential health benefit of participation (*n* = 4, 22.2%), and as a reason to leave the house (*n* = 1, 5.5%).

#### 3.2.2 Reasons for Study Completion

Of the 15 participants who completed the study, 11 provided reasons for remaining in the study. Reasons included commitment (*n* = 8, 72.7%), rapport with study staff (*n* = 4, 36.4%), ease of participation (*n* = 4, 36.4%: exercise as part of a routine, living near the study site, convenient for their schedules and feeling well), and enjoyment of/sense of accomplishment from exercising (*n* = 2, 18.2%). Other reasons included the ability to focus on well‐being (*n* = 1, 6.6%), feeling well enough to attend (*n* = 1, 6.6%), support from their partner (*n* = 1, 6.6%), and flexibility of the intervention schedule (*n* = 1, 6.6%).

#### 3.2.3 Reasons for Study Withdrawal

Three participants were non‐completers, and cited distance from the study site (*n* = 2, 66.7%) and lack of energy/motivation (*n* = 2, 66.7%) as reasons for withdrawal. Other reasons included a lack of resources to exercise at home (*n* = 1, 33.3%) and a lack of understanding of the technology (FitBit) (*n* = 1, 33.3%).

### 3.3 Experience With Study Personnel

Seventeen participants (88.9%) shared feedback regarding study staff, with nearly all respondents (*n* = 16, 94.1%) reporting they were highly satisfied with the study personnel. For example, one participant called the study personnel “the best part of the study” (11P‐Y), and another participant reported that personnel went the “extra 10 miles” (18H‐Y). Participants specifically liked the amount and frequency of interactions with the study personnel (*n* = 10, 58.8%), though one participant reported they would prefer less frequent contact. Participants also reported they liked the study staff's empathic, encouraging, uplifting, and motivating nature (*n* = 7, 41.2%). Study staff were also considered nice, friendly, knowledgeable, and professional (*n* = 10, 58.8%). Participants felt the study staff built a sense of personal connection with participants and between study participants (*n* = 5, 29.4%), that the study staff were attentive and responsive (*n* = 2, 11.7%), and that the study team was flexible (*n* = 1, 5.8%). One participant (5.8%) expressed there were some technical issues with communicating with staff and that they preferred to communicate via email instead of over text message.

### 3.4 Experience in the Study Interventions

Data are organized based on the study intervention group (PAI or HLI). Explanatory quotes are in Table 2.

#### 3.4.1 Physical Activity Intervention (PAI)

##### 3.4.1.1 Expectations Met

Seven of the nine participants (77.8%) who completed PAI provided data on whether their participation met their expectations. Of those, six (85.7%) reported that the PAI intervention met their expectations. Reasons the PAI met expectations included close monitoring throughout the study (*n* = 4, 57.1%) and that the program motivated them to be more active (*n* = 3, 42.9%). One participant expressed that their expectations were unmet because they preferred in‐person exercise and more interaction with the interventionists, but they understood this was impossible due to the remote pivot of exercise during the COVID‐19 pandemic.

##### 3.4.1.2 Perceptions of the Program

Positive aspects of the PAI intervention included the supervised format (*n* = 2, 28.5%), the inclusion of both aerobic and resistance exercise (*n* = 2, 28.5%), and the use of Fitbit (*n* = 2, 28.5%). Two participants (28.5%) indicated that the study helped them get in better shape, two participants (28.5%) liked that the program motivated them, and one (14.2%) reported they liked that they felt better after exercising. Other positive aspects included the “personalized” exercise sessions (*n* = 1, 14.2%), access to a new FitBit (*n* = 1, 14.2%), interaction with other participants (*n* = 1, 14.2%), and access to exercise videos so they could go at their own pace (*n* = 1, 14.2%). Although one participant (14.2%) liked that their blood pressure and heart rate were monitored during the session, another participant (14.2%) reported that this monitoring was cumbersome. Negative aspects included having to exercise virtually (*n* = 3, 42.8%), difficulty accessing online exercise videos (*n* = 1, 14.2%), and the use of FitBit because they preferred more advanced technology for cardiac monitoring (*n* = 1, 14.2%). One participant (14.2%) mentioned a drawback to interacting with other participants during exercise sessions was that it could be uncomfortable to hear about others' experiences when they were worse than their own. Participants suggested that the program would improve if there were a satellite exercise location (*n* = 1, 14.2%), if sessions were held in person (*n* = 1, 14.2%), and if they could access another exercise tracking device (e.g., Apple Watch) (*n* = 1, 14.2%).

Participants also described the barriers and facilitators to completing exercises at home. Barriers included health conditions or side effects from chemotherapy treatments (*n* = 5, 71.4%), lack of proper space/equipment at home (*n* = 4, 57.1%), and lack of motivation (*n* = 1, 14.2%). Two participants (28.5%) felt they could continue their regular exercise routine, making participating in PALS easy. Facilitators included the positive impact on their health (*n* = 2, 28.5%) and intrinsic or extrinsic motivation from family or study team (*n* = 5, 71.4%).

#### 3.4.2 Healthy Living Intervention (HLI)

##### 3.4.2.1 Expectations Met

Five of the six participants (83.3%) who completed HLI provided data on whether their participation met their expectations. Of those, four (*n* = 4; 80.0%) reported that the HLI program met their expectations, with one participant further emphasizing the program “exceeded their expectations” (18H‐Y). The following reasons indicate why their expectations were met: HLI provided a physical and mental outlet following treatment (*n* = 1, 20.0%), HLI had educational content (*n* = 1, 20.0%), and because of community building (*n* = 1, 20.0%). One participant (20.0%) reported they did not have any expectations when enrolling in the study.

##### 3.4.2.2 Perceptions of the Program

All HLI participants provided data on their perceptions of the HLI program, of which, all endorsed positive perceptions (*n* = 6, 100%). Participants liked the content, specifically the holistic nature of the program (*n* = 1, 16.6%), tips for combating cancer side effects (*n* = 1, 16.6%), nutrition information (*n* = 2, 33.3%), and mental health resources (*n* = 2, 33.3%). Participants also enjoyed connecting with other participants through the sessions (*n* = 4, 66.7%). Other reasons included the fact that the content was helpful at a stressful time (*n* = 1, 16.6%), the proximity to the program when held in person (*n* = 1, 16.6%), and the ability to invite their significant other (*n* = 1, 16.6%). One participant (16.6%) liked attending the intervention online, but three others preferred to attend in‐person sessions. One participant (16.6%) reported hearing problems, which made the Zoom sessions challenging. Suggestions for the future included allowing caregivers to attend online sessions (*n* = 1, 16.6%) (Note. Caregivers were permitted to attend in‐person sessions, but not virtual sessions) and adding content on accepting and coping with a cancer diagnosis (*n* = 1, 16.6%).

## 4 Discussion

The inclusion of qualitative interviews during the PALS pilot trial yielded valuable insights to optimize intervention adherence and retention, ultimately to optimize outcomes in the subsequent larger RCT. Results from qualitative interviews of breast cancer and lymphoma patients who participated in the PALS pilot trial and were randomized to a PAI or HLI intervention informed the tailoring of both study conditions for the subsequent, larger RCT designed to evaluate the effects of an exercise intervention on VO_2 peak_ among breast cancer and lymphoma patients undergoing cardiotoxic chemotherapy. The findings highlight important considerations for enrolling and retaining breast cancer and lymphoma patients in clinical exercise studies. Participants overwhelmingly indicated a desire to give back (i.e., altruism) and provider recommendation as the main reasons for enrollment. Reasons patients completed the study included intrinsic motivation, ease of participation, and positive experiences in the study interventions. Overall, feedback on the PAI and HLI interventions was positive, and participants appreciated the empathetic, knowledgeable, and motivating study staff, felt the content was relevant and interesting, and valued that the exercise intervention was tailored to their individual needs. Barriers to intervention adherence included treatment‐related symptom burden and a lack of motivation. Motivation was also cited as a facilitator for adherence.

Our qualitative findings informed modifications to the PAI intervention for the subsequent larger RCT, including greater individualization and ensuring study staff maintain regular communication with participants. Given the qualitative findings that participants appreciated the supervised nature of PAI, even when delivered virtually due to the COVID‐19 pandemic, one modification includes allowing participants a choice between in‐person and virtual supervised sessions moving forward. We will also tailor the PAI to allow for flexibility in the exercise prescription based on participants' symptom burden to address the commonly reported barrier to PA engagement (i.e., due to symptoms). For example, if a participant reports feeling fatigued at the beginning of an exercise session, the interventionist will decrease the prescribed intensity and duration of that day's session. Should participants report that their symptom burden is too high to exercise, the session will be rescheduled, and the interventionist will encourage the participant to complete a home‐based exercise session later that week. In addition to allowing flexibility in PAI, maintaining regular, positive communication with participants will be emphasized for the next phase of PALS. Participants expressed that their interaction with the study staff was uplifting and deemed them pivotal contributors to their overall enjoyment of the study.

The positive feedback regarding HLI suggests it was an acceptable control for the PALS study, and few modifications were needed. Participants particularly liked the content of HLI (e.g., mental health, nutrition) and the opportunity to build relationships with others (e.g., study team, other participants). Although participants varied on their preferences for virtual or in‐person delivery, all participants were willing and able to engage in the online sessions and found them beneficial. Given that participants value interacting with other survivors, we will continue to deliver the HLI content online, allowing participants from both study sites to attend together and allowing for greater attendance and community building. It is also important to state that the HLI content likely did not influence the primary efficacy outcome of the PALS study (i.e., exercise capacity, cardiovascular function), though it may have influenced secondary efficacy outcomes (e.g., quality of life). Offering an alternative intervention (e.g., health workshops) instead of no‐intervention control groups (e.g., usual care, waitlist) can improve the acceptability of randomization [22], particularly for participants from medically underserved populations [23]. This approach may therefore contribute to successful recruitment and retention rates.

Recruiting and enrolling cancer patients for exercise clinical trials can be challenging [18]. The demands of the clinical trial must compete with the timing, side effects, and psychosocial demands of cancer treatment. At the onset of diagnosis, cancer patients can be overwhelmed with the uncertainty of prognosis, treatment decision‐making, and the onslaught of appointments and changes that occur in the immediate days and weeks post‐diagnosis. Given this sensitive period, it is critical to be thoughtful when designing an exercise clinical trial and approaching patients for enrollment. Similar to what was found in the present study, many cancer patients and survivors feel a sense of altruism, which has been shown to be the top reason for enrolling in cancer‐related clinical trials [24]. In addition, it is critical to collaborate with clinicians to both ensure the appropriateness and safety of exercise for patients and to facilitate participant recruitment. Since half of our sample indicated provider recommendation as a reason for enrolling in the PALS study, oncology providers play an essential role in identifying and recommending their patients for clinical exercise trials. Based on this finding, the lymphoma providers who refer patients to the study were invited to join weekly study team meetings to facilitate collaboration. The study team, collaboratively with referring providers, also developed a recruitment script and general talking points of potential participant benefits for providers and research staff to emphasize when patients were approached.

Ease of participation also emerged as an important factor related to study retention and exercise adherence. For example, living close to the study site and lack of symptoms made participating in exercise easier for participants. It is important to note that there was variability in patient preferences for in‐person versus virtual PAI sessions. Some participants indicated they benefited from social support and accountability of in‐person sessions. In contrast, others said they preferred virtual sessions and appreciated home‐based practice for convenience and out of fear of COVID. Suggestions to promote the ease of participating in clinical exercise trials include assessing barriers to PA adherence and developing plans to overcome these barriers, providing compensation for their participation in the trial, providing at‐home exercise equipment, and allowing for flexible intervention delivery (e.g., in‐person, virtually, home‐based) to accommodate individual needs and preferences.

The need for flexible and individualized interventions warrants further discussion. Given the known challenges with symptoms during chemotherapy treatment [19], the exercise intervention was specifically designed to accommodate fluctuation of symptoms [21]. Incorporating this nonlinear approach is essential to promoting exercise adherence among cancer survivors during treatment. Adherence to exercise programming is vital to ensure participants receive the proper dose needed to facilitate physiological adaptation and reach targeted outcomes (e.g., better cardiovascular health, better quality of life). The present findings demonstrate that participants liked that the exercise was flexible and tailored to them, a finding that is not unique to our sample [25]. Furthermore, participant preferences for intervention delivery (virtual vs. in‐person) varied in our study, further supporting the need for flexible interventions.

Acknowledging the tension between intervention fidelity and rigor and flexible interventions is important. Intervention fidelity is critical to understanding intervention efficacy and requires a tightly controlled environment to avoid introducing potential confounding factors. Supervised exercise in a center‐based environment maximizes intervention rigor and maintains intervention control. Direct delivery and observation of exercise components (i.e., frequency, intensity, type, time) ensure that participants receive the prescribed dose or volume of exercise intended. This, however, can come at a high burden to participants due to the time and monetary costs of participating, which can unintentionally exclude participants from medically underserved populations who already have lower access to medical and financial resources. Exercise interventions need to be thoughtfully designed to account for the patient's sociocultural context and overcome barriers such as transportation, access to green space, etc. The PAI program in the PALS study provides one example of how to rigorously offer flexibility in intervention delivery (in‐person, virtual, home‐based) and timing of the intervention (time of day, day of week) while creating individualized exercise programs that account for fluctuations in treatment side effects.

Although flexibility and personalization are essential aspects of exercise intervention design, it is also important to consider how to best capture true intervention effects. Offering flexibility may allow more factors to influence the outcome, such as mode of delivery (in‐person vs. home‐based). It is important to select an appropriate control group that controls for such factors and is attractive to participants. The PALS study utilized an HLI intervention designed to benefit participants but was not expected to influence the primary outcome (i.e., cardiovascular health, cardiorespiratory fitness). HLI was well‐liked and valuable for participants and participants maintained exceptional adherence (87.5%), suggesting HLI is an effective control intervention.

In addition to controlling for confounding factors, measuring the actual dose of exercise participants completed is critical. This requires comprehensive measurement of the frequency (days/week), intensity, time (min/day), and types of exercise that participants complete throughout the entirety of the intervention. Technology advancements have made capturing exercise behavior easier through industry (e.g., ActiGraph, activPAL) and commercial (e.g., Fitbit, Apple) devices. Our team will utilize FitBits and Trainerize to provide a close to real‐time indication of participants' engagement with their prescribed program and to alert trainers when adherence is dropping either due to the impact of symptoms during treatment or for other reasons. This real‐time data can then facilitate modifications to subsequently re‐engage participants in the intervention.

Strengths and limitations should be noted. Strengths of this study include the focus on understanding participant experiences directly from the participant. In addition, interviews were conducted by trained qualitative researchers who were not involved in the delivery of the study interventions. Together, this allowed us to minimize positive bias in participant responses and gather critical information to facilitate the success of the next phase of PALS. There are, however, some limitations. Interviews were conducted over the phone, and there was some distraction and background noise when conducting the interviews. PALS opened before and continued to recruit during the global COVID‐19 pandemic. The experiences of study participants are specific to the unique historical context of this global event. There was wide variability in the time since PALS participation, which introduces some recall bias into our data.

## 5 Conclusion

Implementing cancer exercise trials can be challenging, as cancer survivors face many barriers to engaging in exercise (e.g., overwhelming feelings from a new cancer diagnosis, treatment‐related symptoms). Additionally, such trials require significant resources (e.g., collaboration across disciplines, cancer‐specific exercise expertise). Our results indicate that positive study staff‐patient interactions, delivering tailored and flexible interventions, and utilizing technology to maintain intervention fidelity are effective strategies for ensuring successful implementation and positive participant experiences during clinical exercise trials. Such trials are integral to investigating effective interventions to mitigate the long‐term cardiotoxic effects of chemotherapy and to improve overall health and well‐being of cancer survivors.

## Author Contributions

**Brianna N. Leitzelar:** conceptualization (supporting), validation (equal), visualization (lead), writing – original draft (lead). **Juliana V. Costa:** data curation (equal), investigation (supporting), project administration (equal), writing – original draft (supporting). **Aylin A. Aguilar:** data curation (lead), formal analysis (lead), investigation (lead), methodology (equal), project administration (equal), validation (equal), writing – original draft (supporting), writing – review and editing (equal). **Brianna R. Wolle:** project administration (supporting), writing – original draft (supporting). **Alexander R. Lucas:** conceptualization (equal), funding acquisition (equal), methodology (equal), supervision (equal), validation (equal), writing – review and editing (equal). **Alexandra Marshall:** investigation (supporting), project administration (supporting), validation (equal), writing – review and editing (equal). **Sam Norton:** project administration (equal), validation (equal), writing – review and editing (equal). **Shannon L. Mihalko:** conceptualization (equal), methodology (equal), supervision (equal), validation (equal), writing – review and editing (equal). **Peter H. Brubaker:** conceptualization (equal), funding acquisition (equal), supervision (equal), validation (equal), writing – review and editing (equal). **Rakhee Vaidya:** resources (equal), validation (equal), writing – review and editing (equal). **Mary Beth Seegars:** resources (equal), validation (equal), writing – review and editing (equal). **Victor Yazbeck:** resources (equal), validation (equal), writing – review and editing (equal). **R. Lee Franco:** conceptualization (equal), methodology (equal), supervision (equal), writing – review and editing (equal). **Jeremy Via:** investigation (equal), project administration (equal), validation (equal), writing – review and editing (equal). **W. Gregory Hundley:** conceptualization (equal), funding acquisition (equal), methodology (equal), resources (equal), supervision (equal), validation (equal), writing – review and editing (equal). **Lynne I. Wagner:** conceptualization (equal), methodology (lead), resources (equal), validation (equal), writing – original draft (equal).

## Funding

This work was supported by the National Cancer Institute (Grant number CA226960) and the Qualitative and Patient‐Reported Outcomes Shared Resource of the Atrium Health Wake Forest Baptist Comprehensive Cancer Center's NCI Cancer Center Support Grant P30CA12197. Author B.N.L. was supported in part by a T32 training grant from the National Cancer Institute (CA122061).

## Ethics Statement

All procedures performed in studies involving human participants were in accordance with the ethical standards of the institutional and/or national research committee and with the 1964 Helsinki declaration and its later amendments or comparable ethical standards. Approval was granted by the Wake Forest University Institutional Review Board (IRB00020968).

## Consent

Informed consent was obtained from all individual participants included in the study. The authors affirm that human research participants provided informed consent for publication of individual qualitative data.

## Conflicts of Interest

Author L.I.W. received personal fees unrelated to the present manuscript from Bristol Myers Squibb/Celgene for consultation on patient‐reported outcome/quality of life analysis as a member of the Connect Multiple Myeloma Scientific Steering Committee. The authors have no other relevant financial or non‐financial interests to disclose.

## Data Availability

De‐identified data from this study may be available on reasonable request and with appropriate ethical approvals.
